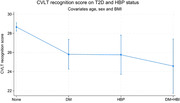# Impact of Type 2 Diabetes and Hypertension on Cognition: A Propensity Score‐Matched Analysis in Mexican Americans from San Antonio

**DOI:** 10.1002/alz.091135

**Published:** 2025-01-09

**Authors:** Juan C Lopez‐Alvarenga, Marcio A Almeida, José E. Cavazos, Michael C Mahaney, Gladys E. Maestre, David C Glahn, Ravindranath Duggirala, John Blangero

**Affiliations:** ^1^ The university of Texas Rio Grande Valley ‐ Edinburg, TX, Edinburg, TX USA; ^2^ University of Texas Rio Grande Valley, Brownsville, TX USA; ^3^ Neurology and Physiology UT Health San Antonio, San Antonio, TX USA; ^4^ Department of Neurosciences and Department of Human Genetics, University of Texas Rio Grande Valley School of Medicine, Brownsville, TX USA; ^5^ South Texas Alzheimer’s Disease Research Center, Harlingen, TX USA; ^6^ University of Texas Rio Grande Valley School of Medicine, Brownsville, TX USA; ^7^ Laboratory of Neuroscience, University of Zulia, Maracaibo, Zulia Venezuela (Bolivarian Republic of); ^8^ RGV Alzheimer’s Center (AD‐RCMAR), Brownsville, TX USA; ^9^ Harvard Medical School, Boston, MA USA; ^10^ Boston Children’s Hospital, Boston, MA USA; ^11^ Texas A&M University, San Antonio, TX USA

## Abstract

**Background:**

Observational studies have reported cognitive domain alterations in individuals with Type 2 diabetes mellitus (T2D), affecting cognitive domains of information‐processing speed, memory, attention, and executive function. Epidemiological observational studies have shown an association between higher blood pressure [HBP], low processing speed, short‐term memory and learning, and delayed recall. Within the US Hispanic/Latino population, specifically Mexican Americans, cognition remains insufficiently studied. This preliminary analysis aims to discern the impact of T2D and hypertension on cognition, employing propensity score matching to mitigate confounders.

**Method:**

A dataset with 650 participants without dementia (mean age 50 ± 12.6 years, BMI 29.1 ± 6.6, 63% females) from the Mexican American Family Study Cohort from low economic strata was analyzed. The effects of T2D and HBP were evaluated using propensity score matching, specifically employing nearest‐neighbor matching under a logistic model. Coefficients (95% confidence intervals) and p‐values were reported.

**Result:**

Among the participants, 9% had T2D, 6% had HBP, and 2% had both conditions. Adjusting for sex, age, and BMI, the presence of T2D impacted face memory delay [b = 1.3 (0.1, 2.5) p = 0.032] and Continuous Performance Test (CPT) hits [b = 45.9 (24.7, 67.1) p<0.001]. Hypertension influenced California Verbal Learning Test (CVLT) learning [b = ‐11.1 (‐15.1, ‐7.4) p<0.001], CVLT recognition [b = ‐3.2 (‐6.1, ‐0.3) p = 0.03], and CPT hits [b = ‐31.1 (‐64.4, 2.3) p = 0.068)—Figure 1 CVLT score on T2D and HBP status.

**Conclusion:**

This preliminary study underscores the heightened risk of hypertension and T2D impacting cognitive function among Mexican Americans. Variations in cognitive measurements based on T2D or hypertension were observed, emphasizing the need for specific cognitive screening tools tailored to the prevalence of chronic conditions in this susceptible population.